# Supplementary material for: A Critical View of the Application of the APEX Software (Aqueous Photochemistry of Environmentally-Occurring Xenobiotics) to Predict Photoreaction Kinetics in Surface Freshwaters
Source: Molecules. 2019 Dec 18;25(1):9. doi: 10.3390/molecules25010009 (PMC7017383; doi:10.3390/molecules25010009)
Supplement: Supplementary file 1 [file molecules-25-00009-s001.zip › 3.2.4_gcc-4.4.0.zip!/3.2.4_gcc-4.4.0/3.2.4_gcc-4.4.0/doc/octave/html/interpreter/Expressions-Involving-Diagonal-Matrices.html]

Expressions Involving Diagonal Matrices - Untitled


Next: Expressions Involving Permutation Matrices,
Up: Matrix Algebra


---

#### 20.2.1 Expressions Involving Diagonal Matrices

Assume D is a diagonal matrix. If M is a full matrix,
then `D*M` will scale the rows of M. That means,
if `S = D*M`, then for each pair of indices
i,j it holds

```
     S(i,j) = D(i,i) * M(i,j).
```

Similarly, `M*D` will do a column scaling.

The matrix D may also be rectangular, m-by-n where `m != n`.
If `m < n`, then the expression `D*M` is equivalent to

```
     D(:,1:m) * M(1:m,:),
```

i.e., trailing `n-m` rows of M are ignored. If `m > n`,
then `D*M` is equivalent to

```
     [D(1:n,n) * M; zeros(m-n, columns (M))],
```

i.e., null rows are appended to the result.
The situation for right-multiplication `M*D` is analogous.

The expressions `D \ M` and `M / D` perform inverse scaling.
They are equivalent to solving a diagonal (or rectangular diagonal)
in a least-squares minimum-norm sense. In exact arithmetics, this is
equivalent to multiplying by a pseudoinverse. The pseudoinverse of
a rectangular diagonal matrix is again a rectangular diagonal matrix
with swapped dimensions, where each nonzero diagonal element is replaced
by its reciprocal.
The matrix division algorithms do, in fact, use division rather than
multiplication by reciprocals for better numerical accuracy; otherwise, they
honor the above definition. Note that a diagonal matrix is never truncated due
to ill-conditioning; otherwise, it would not be much useful for scaling. This
is typically consistent with linear algebra needs. A full matrix that only
happens to be diagonal (an is thus not a special object) is of course treated
normally.

Multiplication and division by diagonal matrices works efficiently also when
combined with sparse matrices, i.e., `D*S`, where D is a diagonal
matrix and S is a sparse matrix scales the rows of the sparse matrix and
returns a sparse matrix. The expressions `S*D`, `D\S`, `S/D` work
analogically.

If D1 and D2 are both diagonal matrices, then the expressions

```
     D1 + D2
     D1 - D2
     D1 * D2
     D1 / D2
     D1 \ D2
```

again produce diagonal matrices, provided that normal
dimension matching rules are obeyed. The relations used are same as described above.

Also, a diagonal matrix D can be multiplied or divided by a scalar, or raised
to a scalar power if it is square, producing diagonal matrix result in all cases.

A diagonal matrix can also be transposed or conjugate-transposed, giving the expected
result. Extracting a leading submatrix of a diagonal matrix, i.e., `D(1:m,1:n)`,
will produce a diagonal matrix, other indexing expressions will implicitly convert to
full matrix.

Adding a diagonal matrix to a full matrix only operates on the diagonal elements. Thus,

```
     A = A + eps * eye (n)
```

is an efficient method of augmenting the diagonal of a matrix. Subtraction works
analogically.

When involved in expressions with other element-by-element operators, `.*`,
`./`, `.\` or `.^`, an implicit conversion to full matrix will
take place. This is not always strictly necessary but chosen to facilitate
better consistency with matlab.
